# Beta burst-driven adaptive deep brain stimulation for gait impairment and freezing of gait in Parkinson’s disease

**DOI:** 10.1093/braincomms/fcaf266

**Published:** 2025-07-09

**Authors:** Kevin B Wilkins, Matthew N Petrucci, Emilia F Lambert, Jillian A Melbourne, Aryaman S Gala, Pranav Akella, Laura Parisi, Chuyi Cui, Yasmine M Kehnemouyi, Shannon L Hoffman, Sudeep Aditham, Cameron Diep, Hannah J Dorris, Jordan E Parker, Jeffrey A Herron, Helen M Bronte-Stewart

**Affiliations:** Department of Neurology and Neurological Sciences, Stanford University School of Medicine, Stanford, CA 94304, USA; Department of Neurology and Neurological Sciences, Stanford University School of Medicine, Stanford, CA 94304, USA; Department of Bioengineering, Stanford Schools of Engineering & Medicine, Stanford, CA 94305, USA; Department of Neurology and Neurological Sciences, Stanford University School of Medicine, Stanford, CA 94304, USA; Department of Neurology and Neurological Sciences, Stanford University School of Medicine, Stanford, CA 94304, USA; Department of Neurology and Neurological Sciences, Stanford University School of Medicine, Stanford, CA 94304, USA; Department of Neurology and Neurological Sciences, Stanford University School of Medicine, Stanford, CA 94304, USA; Department of Neurology and Neurological Sciences, Stanford University School of Medicine, Stanford, CA 94304, USA; Department of Neurology and Neurological Sciences, Stanford University School of Medicine, Stanford, CA 94304, USA; Department of Neurology and Neurological Sciences, Stanford University School of Medicine, Stanford, CA 94304, USA; Department of Bioengineering, Stanford Schools of Engineering & Medicine, Stanford, CA 94305, USA; Department of Neurology and Neurological Sciences, Stanford University School of Medicine, Stanford, CA 94304, USA; Department of Neurology and Neurological Sciences, Stanford University School of Medicine, Stanford, CA 94304, USA; Department of Neurology and Neurological Sciences, Stanford University School of Medicine, Stanford, CA 94304, USA; Department of Neurology and Neurological Sciences, Stanford University School of Medicine, Stanford, CA 94304, USA; Department of Neurology and Neurological Sciences, Stanford University School of Medicine, Stanford, CA 94304, USA; Department of Psychology, University of California Los Angeles, Los Angeles, CA 90095, USA; Department of Neurological Surgery, University of Washington, Seattle, WA 98104, USA; Department of Neurology and Neurological Sciences, Stanford University School of Medicine, Stanford, CA 94304, USA; Department of Neurosurgery, Stanford University School of Medicine, Stanford, CA 94305, USA

**Keywords:** closed loop, local field potential, kinematics, subthalamic nucleus, basal ganglia

## Abstract

Freezing of gait is a debilitating symptom of Parkinson’s disease that is often refractory to medication. Prolonged beta bursts within the subthalamic nucleus are associated with worse impairment and freezing, which are improved with deep brain stimulation. The goal of the study was to investigate the feasibility, safety and tolerability of beta burst-driven adaptive deep brain stimulation for gait impairment and freezing of gait in Parkinson’s disease. Seven individuals with Parkinson’s disease were implanted with the investigational Summit™ RC + S deep brain stimulation system (Medtronic, PLC, Dublin, Ireland). A PC-in-the-loop architecture adjusted stimulation in real-time based on beta burst durations in the subthalamic nucleus. A rigorous calibration procedure was employed to find participant-specific adaptive deep brain stimulation parameters. In a double-blind design, participants performed a harnessed stepping-in-place task, a free walking turning and barrier course, instrumented measures of bradykinesia and clinical motor assessments in four conditions: OFF stimulation, on adaptive, continuous or randomly adapting deep brain stimulation. Adaptive deep brain stimulation was successfully implemented and deemed safe and tolerable in all participants. Gait metrics such as overall percent time freezing and mean peak shank angular velocity improved on adaptive deep brain stimulation compared to OFF and showed similar efficacy as continuous deep brain stimulation. Similar improvements were also seen for overall clinical motor impairment, including tremor and quantitative metrics of bradykinesia. The current pilot study demonstrated initial safety, tolerability, and feasibility of adaptive deep brain stimulation for freezing of gait in Parkinson’s disease in the acute laboratory setting, supporting the future investigation of its longer-term efficacy in the at-home setting.

## Teaser

Wilkins *et al*. report on the safety, feasibility and tolerability of adaptive deep brain stimulation for freezing of gait in Parkinson’s disease using a novel beta burst-driven controller. Stimulation was safe and tolerable in all participants and provided significant improvement in all motor symptoms compared to no stimulation.

## Graphical Abstract

**Graphical Abstract:**
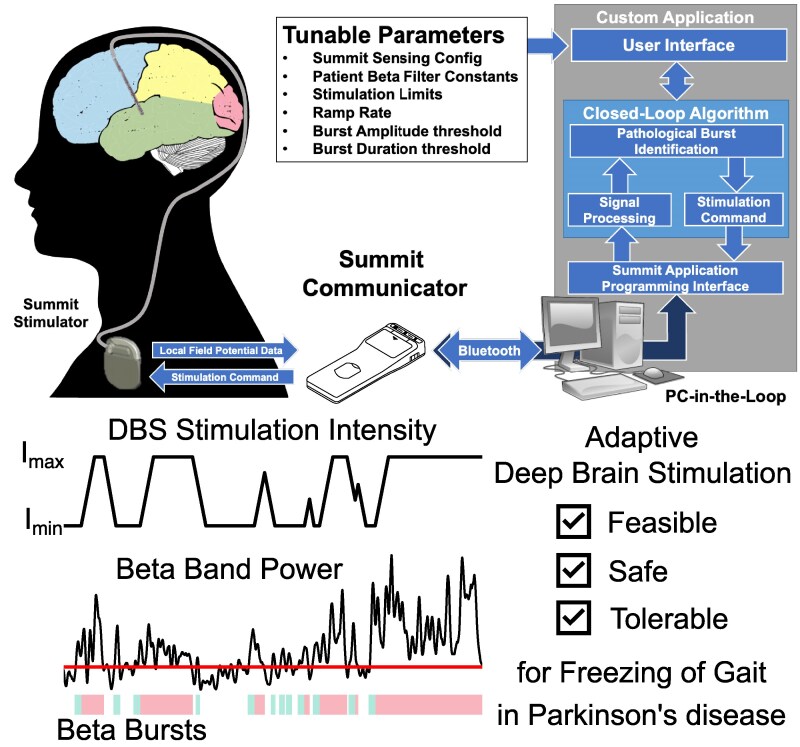

## Introduction

Gait impairment and freezing of gait (FOG) can lead to falls, injury and loss of independence for individuals with Parkinson’s disease.^1,2^ Despite the efficacy of dopaminergic medication for symptoms such as bradykinesia and rigidity, gait impairment and FOG may be refractory to medication.^3,4^ Traditional continuous deep brain stimulation (cDBS) improves gait impairment and FOG to a certain degree in individuals with Parkinson’s disease who are responsive to medication,^5^ but many still experience these symptoms on DBS despite effective treatment of other symptoms, and often gait continues to decline over time.^6-9^ Therefore, there is a growing need for novel therapies that may better target gait impairment and FOG in Parkinson’s disease.

Subcortical local field potential (LFP) recordings from the subthalamic nucleus (STN) revealed a potential pathological neural feature of FOG in Parkinson’s disease.^5^ Freezers showed prolonged beta band (13–30 Hz) burst durations compared to non-freezers in the STN, which further increased during episodes of FOG. DBS acted to shorten these pathologically long beta bursts, and improved FOG. Considering beta burst duration both relates to the observed impairment of FOG and is also modulated by DBS intensity alongside improvements in behaviour, it offers the potential to serve as a relevant neural input for adaptive DBS (aDBS) in which stimulation amplitude is adjusted in real-time based on a given input.^5^ The majority of aDBS studies have been implemented either at rest, during seated tasks, or in patients who did not exhibit FOG, and therefore it is unknown how aDBS may affect gait impairment and FOG. An initial case study using the first-generation sensing neurostimulator (Activa® PC + S, Medtronic, PLC, Dublin, Ireland) evaluating beta power-driven aDBS found improved gait and reduced FOG compared to OFF DBS and traditional cDBS in one participant based on quantitative kinetics.^10^ However, this device was unable to use beta burst durations as a neural input due to technical limitations and was only in one participant. Similarly, a recent case report found long-term benefit using power-driven aDBS for gait using the AlphaDBS neurostimulator.^11^ There have been no demonstrations using beta burst durations as an aDBS input in the chronic freely moving state, despite its potential as a biomarker.

Advances in DBS technology now enable the possibility of using beta burst durations as a neural input for aDBS in the chronic setting using the investigative Summit™ RC + S neurostimulator (Medtronic, PLC, Dublin, Ireland). The goal of the current study was to investigate the feasibility, safety and tolerability of beta burst-driven aDBS for gait impairment and FOG in Parkinson’s disease in the laboratory acute setting. Adaptive DBS, cDBS and a randomly adapting DBS control condition were tested in a double-blinded, randomized study in the largest cohort of participants implanted with the Summit™ RC + S neurostimulator to date. We performed quantitative assessments of gait impairment and FOG, as well as assessments of overall motor symptoms to evaluate its effect on both gait and other PD symptoms. A bench to bedside model was employed in which adaptive DBS was first assessed during a harnessed gait task for shorter durations of time, before then being evaluated in a free walking environment for longer durations. We hypothesized that beta burst-driven aDBS would be feasible, safe and tolerable in all participants, and provide similar efficacy as continuous DBS.

## Materials and methods

### Human subjects

Seven participants (5 male, 2 female) with clinically established Parkinson’s disease underwent bilateral implantation of DBS leads (model 3389, Medtronic, PLC, Dublin, Ireland) in the sensorimotor region of the STN.^12^ Leads were connected to the investigative neurostimulator Summit™ RC + S which was implanted in the right chest. Inclusion criteria included being at least 18 years of age, meeting criteria for STN DBS,^12^ presence of complications of medication such as wearing off signs, fluctuating responses, dyskinesias, medication refractory tremor and/or impairment in the quality of life on or off medication, and a score ≥ 1 on the freezing of gait questionnaire (FOG-Q) and/or gait sub-score (Item 3.10) of Movement Disorders Society Unified Parkinson’s Disease Rating Scale Part III (MDS-UPDRS III) in the off or on state. Exclusion criteria included being over the age of 80, dementia, untreated psychiatric disease, Hoehn and Yahr stage 5, major surgical morbidities, presence of a cardiac pacemaker, required rTMS, ECT, MRI, or diathermy, pregnancy, cranial metallic implant or history of seizures or epilepsy. The initial planned cohort was 15 individuals, but recruitment was halted after discontinuation of the Summit™ RC + S in October 2022. All participants gave written consent to participate in the study, which was approved by the Food and Drug Administration with an Investigational Device Exemption and by the Stanford University Institutional Review Board.

### Experimental protocol

Experiments occurred after the participant had been deemed clinically optimized on their DBS settings by their clinician. Experimental testing was done in the off-medication state, which entailed stopping long-acting dopamine agonists at least 48 h, dopamine agonists and controlled release carbidopa/levodopa at least 24 h and short-acting medication at least 12 h before testing. The protocol was split into two separate visits separated by 3 months, each consisting of 3 to 6 days of testing. The design of the protocol was a ‘bench to bedside’ model. During the first visit, a harnessed gait task was performed to test the initial safety and tolerability of the aDBS settings for a shorter duration of time. If the first visit was successful, then during the second visit, a free walking task was performed and aDBS was assessed over a longer duration. The details of the two visits are as follows.

Randomized stimulation titrations ranging from 25% to 125% of their clinical stimulation amplitude were performed to determine the therapeutic window and corresponding LFP beta band burst duration threshold for aDBS. Stimulation did not exceed 125% of clinical stimulation as to avoid side effects and ensure participants did not receive excessive stimulation compared to their optimized clinical settings. Following titrations, the participant underwent ramp rate testing to find a tolerable rate for adjusting stimulation during aDBS. The last portion of the calibration testing involved calibration runs of aDBS during rest and gait to test out the determined therapeutic window, ramp rate and aDBS thresholds. See Supplementary Materials for details.

Participants were tested OFF DBS and on three stimulation conditions: cDBS, aDBS and randomly adapting DBS (rDBS). During cDBS stimulation amplitude is held constant, whereas during rDBS stimulation amplitude varies randomly using the same ramp rate and therapeutic window as aDBS. The pattern of rDBS was determined by randomly shuffling the adaption pattern observed during the calibration aDBS runs to maintain similar total electrical energy delivered (TEED) and patterns of adaption but unlinking it with the beta biomarker. The order of DBS conditions was randomized, and the participant was blinded to the condition. All three DBS conditions used the same active contacts, frequency (140.1 Hz) and pulse width (60 μs).

For the first visit, participants completed a 100-s stepping-in-place task and MDS-UPDRS III by a blinded certified rater. The stepping-in-place task is a validated assessment of FOG and involves self-paced alternating stepping on two force plates while harnessed.^13^ Testing was done after a 20-min wash-in of the stimulation condition. For the second visit, participants completed a turning and barrier course, MDS-UPDRS III, and a repetitive wrist flexion-extension task. The turning and barrier course is a validated free walking task designed to elicit FOG and is made up of various dividers that simulate narrow hallways and doorways.^14^ Participants walk in two ellipses in one direction, followed by two figures of eights; they then repeat in the opposite starting direction. The initial direction was randomized. The wrist flexion-extension task is a validated assessment of bradykinesia.^15-18^ During the task, participants flex the forearm so that the elbow is angled at 90° and then flex and extend the hand at the wrist joint as quickly as possible for 30 s. Testing was done after a 60-min wash-in of the stimulation condition and repeated after an additional 60 min (i.e. 120-min timepoint). The OFF condition was only assessed at one timepoint. Supplementary Fig. 1 shows a schematic of the protocol. Testing was considered safe and tolerable if aDBS did not cause adverse effects that resulted in the participant being unable to tolerate the experiment.

### Distributed neural adaptive DBS system

**Figure 1:**
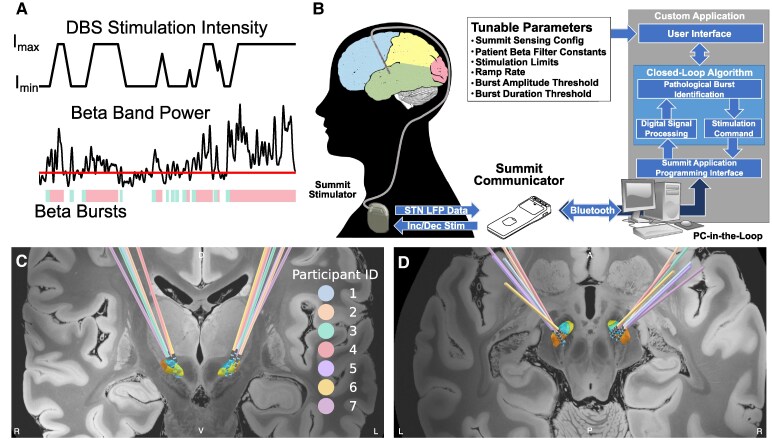
**Beta burst duration control policy algorithm and DBS lead locations within the STN**. Burst durations are calculated from sequential crossings of the envelope of beta power over a participant-specific threshold (horizontal red line) is shown in **A**. Mint bars indicate physiological burst durations and the transition to rosy pink shows when the burst has become pathological (i.e. exceeded the participant-specific burst duration threshold). DBS intensity increases or decreases at tolerable ramp rates within the therapeutic window in response to the beta burst duration. PC-in-the-loop architecture utilizes the Summit communicator to provide STN beta burst-based adaptive stimulation is shown in **B**. Coronal and Axial views of DBS lead locations within the STN for all 7 participants are shown in **C** and **D**, respectively. Inc/Dec, Increment/Decrement Stimulation; INS, Implantable Neurostimulator; LFP, Local Field Potential; STN, Subthalamic Nucleus. Summit Communicator and INS images are both credited to Medtronic. Figure adapted from Petrucci *et al*. ^19^ Reprinted with permission from IEEE Proceedings.

The current study used the Summit™ RC + S DBS system which consists of a rechargeable implanted neurostimulator (INS) with sensing and closed-loop stimulation capabilities, a bidirectional communicator, and a C# application programming interface (API) that allows the creation of custom applications (Fig. 1). The API can be used to configure the INS and perform ‘distributed’ closed loop stimulation where neural data is streamed from the INS via Bluetooth to a PC-in-the-loop. The neural data are analyzed in real-time and commands to change stimulation based on the control policy algorithm are sent back to the INS via the communicator.^19,20^

### Neural adaptive DBS control policy algorithm

A single threshold algorithm based on STN beta burst durations was used, in which stimulation increased when the observed current beta burst duration was longer than a participant-specific threshold, or decreased if it was shorter.^19,21^ LFP data were streamed from each STN and added into two buffers. The LFP data were filtered with a 128-order bandpass FIR filter around a participant-specific 6 Hz band within the beta band (13–30 Hz). The filtered data were squared, and then peaks were identified and then linear interpolated to create a beta envelope. A participant-specific threshold for finding the start/end of a given beta burst was determined based on the average trough (minima) power in the linear envelope of the signal in the 45–65 Hz band from the OFF recording.^21^ The most recent current beta burst was identified by finding the most recent period in the buffered data where the envelope was above the participant-specific threshold. This algorithm is based on the concept of identifying ‘pathological’ bursts in which the observed duration is longer than a ‘normal’ physiological burst, which can be determined based on the participant-specific 1/*f* distribution.^21^ If multiple peaks within the beta frequency range were observed, the peak with the greatest modulation of burst duration with increasing stimulation was chosen. The algorithm was implemented bilaterally if both STNs had usable neural signal, with the decision for each STN occurring independently. If aDBS was only implemented unilaterally, the other hemisphere was held at 100% clinical stimulation amplitude. Changes in stimulation were in increment or decrement steps of 0.1 mA.

### Therapeutic window

The randomized stimulation intensity titrations during the calibration testing were used to determine the range in which stimulation was allowed to adjust within, i.e. the ‘therapeutic window’ for each participant.^22^ The minimum allowed stimulation intensity (i.e. *I*_min_) was established as the minimum stimulation amplitude that provided an acceptable therapeutic benefit to the participant’s gait compared to the OFF condition. The maximum allowed stimulation intensity (i.e. *I*_max_) was established as the maximum stimulation amplitude that did not elicit adverse side effects or lead to a deterioration of gait performance. It did not exceed 125% of the participant’s clinical stimulation amplitude. For tremor dominant individuals, the *I*_min_ was set to a high enough stimulation amplitude in which there was both a therapeutic benefit to gait and observed tremor was largely absent.

### Threshold determination

The threshold for ‘pathological’ beta burst durations was determined on a participant-specific basis. The average observed beta burst duration during the OFF DBS gait task or at *I*_min_ was used as the initial threshold for the first calibration run. The threshold was then adjusted based on a combination of observed stimulation modulation, participant feedback and the observed kinematics during the calibration runs.

### Ramp rate

Tolerability of ramp rate of stimulation intensity was tested through a custom C# application prior to the calibration run of aDBS.^20^ During ramp rate testing, stimulation intensity randomly fluctuated between *I*_min_ and *I*_max_ at a given ramp rate for several minutes while the participant was seated at rest. The participant gave feedback of whether they felt any symptoms or sensations such as tingling (e.g. paresthesia). A 0.1 mA/s ramp up and 0.05 mA/s ramp down was used if it was tolerable for the participant. This slow ramp rate was used due to the presence of artefact in the LFP signal after each increment or decrement of stimulation^23^ (Supplementary Video 1). The 0.1 mA/s ramp rate allowed sufficient time for the artefact to subside and collection of artefact-free data after a change in stimulation before the next decision was made. If the participant was unable to tolerate the 0.1 mA/s ramp up, then the ramp was slowed until a tolerable rate was found. The ramp down was always set to half the rate of ramp up to bias stimulation up.^24^

### Data acquisition and analysis

#### Kinetic and kinematic data acquisition

Ground reactions forces from dual force plates were sampled at 1000 Hz using a Bertec system (Bertec Corporation, Columbus, OH, US) during the stepping-in-place task.

Kinematics were measured during the gait (stepping-in-place and turning and barrier course) and wrist flexion-extension tasks using wearable inertial measurement units (IMUs, APDM, Inc., Portland, OR). Eleven IMU sensors were positioned on the body for the gait tasks: two on the shanks, two on the feet, two on the thigh, one on the lumbar, one on the chest, two on the wrists and one on the forehead. Two IMU sensors were positioned on the dorsum of the hand for the wrist flexion-extension task. 3D angular velocities from the IMUs’ triaxial gyroscope were sampled at 128 Hz.

#### LFP data acquisition

LFP data were streamed using the Summit™ RC + S neurostimulator. Two channels, one from each STN, were streamed at 500 Hz. A 0.5 Hz onboard high pass filter and two 100 Hz onboard low pass filters were used.^25^ Sense-friendly configurations were used, which consisted of flanking the stimulation contacts (0 to 2, 1 to 3, or 0 to 3^22^). If necessary, clinical stimulation contacts were modified to ensure at least one sense-friendly STN configuration. For details on LFP data acquisition, see Supplementary Materials.

#### Kinetic and kinematic data analysis

##### Stepping-in-place

Vertical ground reaction forces under each foot were converted to percentage of body weight and separated into gait cycles (see Supplementary Materials). Swing times and stride times were used to calculate asymmetry and arrhythmicity. Asymmetry was defined as: asymmetry = 100×|ln(average swing time from leg with shortest swing time/average swing time from leg with longest swing time)|. A larger asymmetry value is indicative of more asymmetric gait. Arrhythmicity was defined as the mean stride time coefficient of variation (standard deviation/mean) of both legs. A larger stride time coefficient of variation is indicative of less rhythmic gait. A previously validated computerized algorithm was used to identify episodes of FOG from the force plate data.^13^ Briefly, this algorithm detects instances where the participant’s feet do not fully lift off from the force plates by identifying peaks in the force plate data that fall below 15% and above 85% of the participant’s bodyweight.

##### Turning and barrier course

Angular velocities of the shanks were filtered using a zero-phase eigth order low pass Butterworth filter with a 9 Hz cut-off frequency, and principal component analysis was used to extract the shank angular velocity within the sagittal plane.^14^ The sagittal plane angular velocity was used to define aspects of the gait cycle (see Supplementary Materials). Swing times and stride times were used to calculate asymmetry and arrhythmicity, as defined above for stepping-in-place. Episodes of FOG were identified on a stride-by-stride basis using a previously validated logistic regression model.^14^ The logistic regression freezing model computes the freeze probability over time using arrhythmicity and asymmetry over the last six steps, and stride time and swing angular range from the last step as relevant input parameters. A freeze was indicated when the freeze probability from the logistic regression exceeded 0.7.

##### Repetitive wrist flexion-extension

Angular velocity of each hand in the plane of flexion-extension was low-pass filtered using a zero-phase fourth order Butterworth filter with a 4 Hz cut-off frequency. Root mean square velocity (*V*_rms_) was calculated to quantify bradykinesia.

### Offline neural data analysis

For the offline neural analysis to determine parameters for aDBS, power spectral density (PSD) estimates were calculated using Welch’s method with a 1-s Hanning window and 50% overlap.^26^ PSDs were calculated across the OFF and stimulation titration conditions. Beta bursts were calculated using a 6 Hz band around the peak beta frequency that showed modulation with increasing stimulation intensity.^19,21^

### Statistical analysis

Statistical analyses were run in R (version 3.6.0, R Foundation for Statistical Computing, University of Auckland, New Zealand). Linear mixed effects models with a fixed effect of stimulation amplitude and random effect of participant nested with STN side were run to examine the effect of stimulation amplitude on beta burst durations. Linear mixed effects models with a fixed effect of stimulation condition and random effect of participant were run for each of the behavioural outcomes. Partial eta squared (${\eta_{p}}^{2}$) are reported. Estimated marginal means were computed for post-hoc pairwise comparisons between stimulation conditions with Tukey’s method to control for multiple comparisons. Significance was set at *P* < 0.05.

## Results

### Demographic characteristics

Demographics of the seven individuals with Parkinson’s disease are shown in Table 1. The participants (age: 63.7 ± 4.9 years) had a mean pre-op off therapy MDS-UPDRS III of 37.1 ± 9.4 and a disease duration of 14.0 ± 2.5 years. Six of the seven participants were classified as freezers based on the FOG-Q or new FOG-Q (nFOG-Q) and the other participant showed gait impairment as evident by a score of 1 on the MDS-UPDRS III gait item 3.10. The Summit™ RC + S was the initial IPG in four participants, whereas three participants received it as an IPG replacement. Figure 1C and D visualize the DBS lead locations within the STN for all participants. Participants performed the first visit on average 53.9 ± 32.7 (range: 6–95) months after their Initial Programming and 22.6 ± 9.4 (range: 7–37) months after being implanted with the Summit™ RC + S. The second visit occurred on average 3 ± 1 (range: 2–5) months after the first visit.

**Table 1:** Participant demographics

ID	Age (years)	Sex	MDS-UPDRS III pre-op		FOG-Q	Disease duration (years)	First IPG?
			OFF meds	ON meds			
01	63	M	27	12	0	11	Yes
02	60	M	50	29	9	15	No
03	72	F	42	16	19	18	Yes
04	69	M	46	35	9	14	Yes
05	65	M	39	23	12	10	No
06	58	M	22	10	24^a^	15	Yes
07	59	F	34	6	10^a^	15	No

^a^New FOG-Q (nFOG-Q).

### Calibration of aDBS

**Figure 2:**
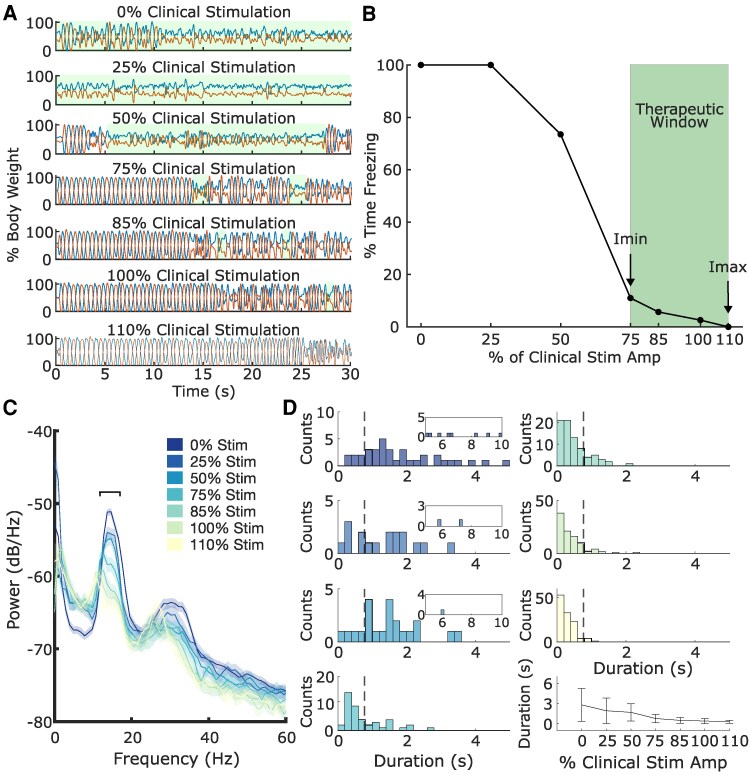
**Example of calibration to determine stimulation limits and neural parameters for neural aDBS.** Performance during the stepping-in-place task at different randomized levels of stimulation amplitude for P03 is shown in **A**. Stimulation amplitude is represented as % of the participant’s clinical stimulation amplitude. Vertical ground reaction forces from the two force plates are depicted as % body weight. Episodes of FOG highlighted in green shading. Percentage of time freezing during the stepping-in-place task at each of the stimulation levels tested with the observed therapeutic window denoted in green is shown in **B**. *I*_min_ and *I*_max_ refer to the minimum and maximum allowed stimulation amplitude during aDBS. PSD plots for different levels of stimulation amplitude during the stepping-in-place task are shown in **C**. Stimulation amplitude is represented as % of the participant’s clinical stimulation amplitude. The chosen 6-Hz band of interest for beta bursts is denoted by the black horizontal line cantered around 15 Hz. Histograms of the observed beta burst durations at the different corresponding levels of stimulation amplitude, with the average and standard deviation of the burst duration across stimulation levels in the lower right are shown in **D**. The corresponding burst duration threshold is shown by the vertical dashed line. dB, decibels; Hz, Hertz; s, seconds.

Figure 2A shows an example of a participant’s stepping-in-place ground reaction force traces at different stimulation amplitudes as part of the calibration testing. The participant is frozen for the entirety of the trial both at 0% (OFF) stimulation and at 25% of their clinical stimulation amplitude, and for the majority of the trial at 50% clinical stimulation. A noticeable improvement is observed at 75% of their clinical stimulation amplitude, which continues to improve with increasing levels of stimulation up to 110% of their clinical stimulation amplitude. The therapeutic window for this participant was set from 75% to 110% of their clinical stimulation amplitude (Fig. 2B).

Figure 2C and D shows the matching neural data for one STN from the behaviour depicted in Fig. 2A and B. A pronounced beta peak is observed around 15 Hz, which attenuates with increasing levels of stimulation intensity. Figure 2D depicts the histograms of the corresponding beta burst durations for the 6-Hz band highlighted in Fig. 2C and the corresponding burst duration threshold shown by the vertical dashed line. A wide distribution of beta burst durations is observed in the OFF DBS condition that continues even at 25% and 50% of the participant’s clinical stimulation amplitude, although to a less extreme extent. The burst durations continue to shorten with increasing stimulation amplitude with most of the bursts having durations under 500 ms at higher stimulation intensities.

Overall, increasing levels of stimulation amplitude led to a significant reduction in beta burst durations during calibration for both the first visit (*t* = 4.16, *P* = 1.4e−4; Supplementary Fig. 2) and second visit (*t* = 3.09, *P* = 4.3e−3) across the participants. For the first visit, four participants used bilateral aDBS and three participants used unilateral aDBS. For the second visit, three participants used bilateral aDBS and three participants used unilateral aDBS. The average ± standard deviation for *I*_min_ was 2.8 ± 1.1 mA (78.1 ± 16.8%) and *I*_max_ was 3.8 ± 1.2 mA (107.8 ± 10.0%) across the cohort. Table 2 shows the individual aDBS parameters used across the participants at each visit.

**Table 2:** Individual aDBS controller parameters

ID	Visit	LSTN contacts	RSTN Contacts	LSTN range (mA)	RSTN range (mA)	LSTN ramp up/down (mA/s)	RSTN ramp up/down (mA/s)	LSTN center freq. (Hz)	RSTN center freq. (Hz)	LSTN burst duration threshold (ms)	RSTN burst duration threshold (ms)
01	SIP	1^−^,2^−^,C^+^	9^−^,C^+^	2.5–4.9	1.8–2.8	0.1/0.05	0.1/0.05	23.4	17.6	225	251
	TBC	1^−^,2^−^,C^+^	9^−^,C^+^	3.6–4.8	2.2–2.9	0.1/0.05	0.1/0.05	18.5	21.5	300	178
02	SIP	1^−^,C^+^	9^−^,C^+^	3.7–4.1	4.1–4.5	0.1/0.05	0.1/0.05	19.5	−*	400	−*
	TBC	1^−^,C^+^	8^−^,9^−^,C^+^	3.7–4	6.4–6.4	0.067/0.033	−	17.6	−^b^	500	−^b^
03	SIP	1^−^,2^−^,C^+^	9^−^,C^+^	2.3–3	3.2–3.2	0.1/0.05	−	14.6	−^a^	771	−^a^
	TBC	1^−^,2^−^,C^+^	9^−^,C^+^	2.3–3.4	3.4–3.4	0.1/0.05	−	13.7	−^a^	852	−^a^
04	SIP	1^−^,2^−^,C^+^	9^−^,10^−^,C^+^	2.6–3.9	5–5	0.1/0.05	−	17.5	−*	270	−*
	TBC	1^−^,2^−^,C^+^	9^−^,10^−^,C^+^	2.7–4.1	5.2–5.2	0.1/0.05	−	18.6	−*	225	−*
05	SIP	1^−^,2^−^,C^+^	9^−^,C^+^	3.4–4.3	3.5–4.5	0.1/0.05	0.1/0.05	14.6	14.6	380	350
	TBC	1^−^,2^−^,C^+^	8^−^,9^−^,C^+^	4.2–5	4.6–5.4	0.1/0.05	0.075/0.0375	16.5	−^b^	390	−^b^
06	SIP	2^−^,C^+^	9^−^,C^+^	2.2–2.8	1.3–1.6	0.1/0.05	0.1/0.05	14.7	14.6	350	400
	TBC	2^−^,C^+^	9^−^,C^+^	1.2–2.4	0.9–1.7	0.1/0.05	0.1/0.05	22.5	25.4	300	254
07	SIP	2^−^,C^+^	9^−^,C^+^	2.6–3.8	4.4–5.9	0.075/0.0375	0.075/0.0375	25.3	13.7	236	333

Dashes indicate instances where an STN was not used for neural recording.

^*^indicates due to stimulation artefact.

^a^indicates due to insufficient modulation of beta burst durations.

^b^indicates due to requiring nonsense friendly configurations for therapeutic efficacy.

STN, subthalamic nucleus; SIP, stepping-in-place (first visit); TBC, turning and barrier course (second visit); C, Case; Hz, Hertz; ms, milliseconds; mA, milliamps.

### Stimulation improves overall clinical motor impairment

**Figure 3:**
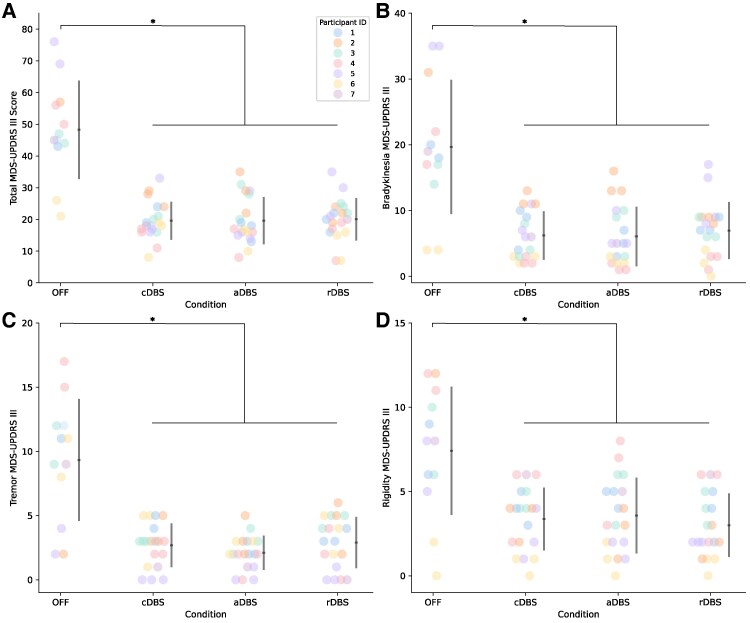
**Overall clinical motor impairment and sub-scores across stimulation conditions.** Total MDS-UPDRS III is shown in **A**, Bradykinesia sub-score is shown in **B**, Tremor sub-score is shown in **C**, Rigidity sub-score is shown in **D**. Individual data are shown, coloured by participant ID, along with the average and standard deviation. Data from all visits and timepoints are included (*N* = 7 participants and 69 total datapoints: OFF = 12, cDBS = 19, aDBS = 19, rDBS = 19; one datapoint per person each for First and Second Visits for OFF DBS and 1 datapoint per person for First Visit and 2 datapoints for Second Visit for ON DBS conditions). Linear mixed effects models found significant effects for all four scores: (Total MDS-UPDRS III) *F*(3,59.3) = 55.8, *P* = 2.2e−16, (Bradykinesia sub-score) *F*(3,59.3) = 40.3, *P* = 2.60e−14, (Tremor sub-score) *F*(3,59.2) = 35.2, *P* = 3.39e−13, and (Rigidity sub-score) *F*(3,59.1) = 20.5, *P* = 3.28e−9. Post-hoc estimated marginal means found significant differences between OFF and DBS conditions for all four scores: (Total MDS-UPDRS III) OFF-cDBS: *t* = 11.2, *P* < 0.0001, OFF-aDBS: *t* = 11.2, *P* < 0.0001, OFF-rDBS: *t* = 11.0, *P* < 0.0001, (Bradykinesia sub-score) OFF-cDBS: *t* = 9.59, *P* < 0.0001, OFF-aDBS: *t* = 9.70, *P* < 0.0001, OFF-rDBS: *t* = 9.09, *P* < 0.0001, (Tremor sub-score) OFF-cDBS: *t* = 8.64, *P* < 0.0001, OFF-aDBS: *t* = 9.39, *P* < 0.0001, OFF-rDBS: *t* = 6.25, *P* < 0.0001, (Rigidity sub-score) OFF-cDBS: *t* = 6.60, *P* < 0.0001, OFF-aDBS: *t* = 6.25, *P* < 0.0001, OFF-rDBS: *t* = 7.21, *P* < 0.0001. * indicates *P* < 0.05.

No adverse events occurred during aDBS that caused the participant to halt testing, thus indicated it was safe and tolerable. Double blinded rated assessments of overall total motor impairment using the MDS-UPDRS III showed a significant effect of stimulation condition (*F*(3,59.3) = 55.8, *P* = 2.2e−16, ${\eta_{p}}^{2}$ = 0.74). Data from both visits were included for each participant. aDBS (*t* = 11.2, *P* < 0.0001; 59.9 ± 15.8% improvement), cDBS (*t* = 11.2, *P* < 0.0001; 58.6 ± 13.8% improvement) and rDBS (*t* = 11.0, *P* < 0.0001; 57.5 ± 11.7% improvement) all showed significant improvements compared to OFF DBS (Fig. 3A). No difference was observed between ON stimulation conditions. One participant was unable to perform the MDS-UPDRS III OFF DBS at the second visit due to inability to tolerate being OFF stimulation.

A significant effect of stimulation condition was seen for each of the sub-scores of the MDS-UPDRS III, including bradykinesia (*F*(3,59.3) = 40.3, *P* = 2.60e−14, ${\eta_{p}}^{2}$ = 0.67), tremor (*F*(3,59.2) = 35.2, *P* = 3.39e−13, ${\eta_{p}}^{2}$ = 0.64) and rigidity (*F*(3,59.1) = 20.5, *P* = 3.28e−9, ${\eta_{p}}^{2}$ = 0.51) (Fig. 3B and D). Data from both visits were included for each participant. aDBS, cDBS and rDBS all showed significant improvements compared to OFF DBS for each of the sub-scores of the MDS-UPDRS III (Supplementary Table 1). No difference was observed between any of the ON-stim conditions for any of MDS-UPDRS III sub-scores (Supplementary Table 2).

### Stimulation improves stepping in harnessed gait task

**Figure 4:**
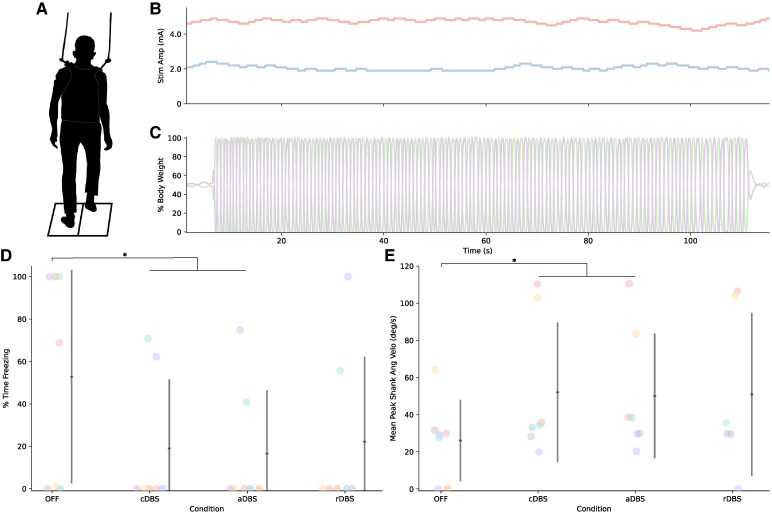
**aDBS during harnessed stepping-in-place task.** Depiction of the harnessed stepping-in-place task is shown in **A**. Example of neural aDBS during stepping-in-place task for one participant (P01) is shown in **B** to **D**. Stimulation adapting independently for both STNs is shown in **B**. The left STN is shown in blue and the right STN in red. Vertical ground reaction forces from the two force plates depicted as % of body weight are shown in **C**. Percent time freezing is shown in **D** and mean peak shank angular velocity during stepping-in-place across stimulation conditions is shown in **E**. Individual data are shown, coloured by participant ID, along with the average and standard deviation (*N* = 7 participants and 28 total datapoints: OFF = 7, cDBS = 7, aDBS = 7, rDBS = 7; one datapoint per person). Linear mixed effects models found significant effects for both metrics: (percent time freezing) *F*(3,18) = 4.53, *P* = 0.016, (mean peak shank angular velocity) *F*(3,17.1) = 4.19, *P* = 0.022. Post-hoc estimated marginal means found significant differences between OFF and DBS conditions for both metrics: (percent time freezing) OFF-cDBS: *t* = 3.01, *P* = 0.035 and OFF-aDBS: *t* = 3.22, *P* = 0.022, (mean peak shank angular velocity) OFF-cDBS: *t* = 3.11, *P* = 0.029 and OFF-aDBS: *t* = 2.88, *P* = 0.047. * indicates *P* < 0.05.

Overall, there was a significant effect of stimulation condition on percent time freezing during stepping-in-place (*F*(3,18) = 4.53, *P* = 0.016, ${\eta_{p}}^{2}$ = 0.42; Fig. 4). Post-hoc pairwise comparisons showed significant reductions in percent time freezing compared to OFF stimulation for aDBS (*t* = 3.22, *P* = 0.022) and cDBS (*t* = 3.01, *P* = 0.035), and a trend for rDBS (*t* = 2.72, *P* = 0.062). No difference was observed between ON stimulation conditions (aDBS–cDBS: *t* = 0.22, *P* = 1.00; aDBS–rDBS: *t* = 0.51, *P* = 0.96; cDBS–rDBS: *t* = 0.29, *P* = 0.99). All five participants who demonstrated freezing in the OFF condition showed improvements in freezing for aDBS and cDBS. In three of those cases, the participant was frozen for the entirety of the trial in the OFF condition and showed substantial improvement with aDBS (25–100% improvement in percent time freezing). The other two participants showed no freezing across any conditions.

There was a significant effect of stimulation condition on mean peak shank angular velocity (*F*(3,17.1) = 4.19, *P* = 0.022, ${\eta_{p}}^{2}$ = 0.42). Post-hoc pairwise comparisons showed significant increase compared to OFF stimulation for aDBS (*t* = 2.88, *P* = 0.047) and cDBS (*t* = 3.11, *P* = 0.029), and a trend for rDBS (*t* = 2.52, *P* = 0.093). No difference was observed between ON stimulation conditions (aDBS–cDBS: *t* = 0.23, *P* = 1.00; aDBS–rDBS: *t* = 0.22, *P* = 1.00; cDBS–rDBS: *t* = 0.44, *P* = 0.97). One datapoint for Participant 2 (rDBS) was missing due to an IMU malfunction.

Three participants (Participants 2, 3, and 4) did not have values for the OFF condition for gait arrhythmicity or asymmetry due to being frozen for the entirety of the stepping-in-place trial. There was no significant effect of stimulation condition on gait arrhythmicity (*F*(3,15) = 1.62, *P* = 0.227, ${\eta_{p}}^{2}$ = 0.239). However, lack of data from the three participants who were frozen for the entirety of the stepping-in-place trial likely masked the effect of stimulation condition on gait arrhythmicity, as all three were able to perform the task when ON stimulation. All four participants who were able to step during the OFF condition showed improvements in arrhythmicity for aDBS compared to OFF stimulation. There was a significant effect of stimulation condition on asymmetry (*F*(3,15) = 7.27, *P* = 0.0031, ${\eta_{p}}^{2}$ = 0.59). Post-hoc pairwise comparisons showed significant reductions in asymmetry compared to OFF stimulation for aDBS (*t* = 4.59, *P* = 0.0018), cDBS (*t* = 3.57, *P* = 0.013) and rDBS (*t* = 3.55, *P* = 0.014). No difference was observed between ON stimulation conditions (aDBS–cDBS: *t* = 1.25, *P* = 0.61; aDBS–rDBS: *t* = 1.28, *P* = 0.59; cDBS–rDBS: *t* = 0.026, *P* = 1.00). Individual data are included in Supplementary Table 3.

### Stimulation improves gait in free walking task

Two participants did not perform the turning and barrier course. Participant 5 did not perform the turning and barrier course due to inability to safely perform the task without assistance under clinical stimulation. They still partook in all other aspects of the visit (i.e. MDS-UPDRS III and wrist flexion-extension task). Participant 7 did not take part in the visit due to stopping their participation in the trial early for unrelated personal reasons.

**Figure 5:**
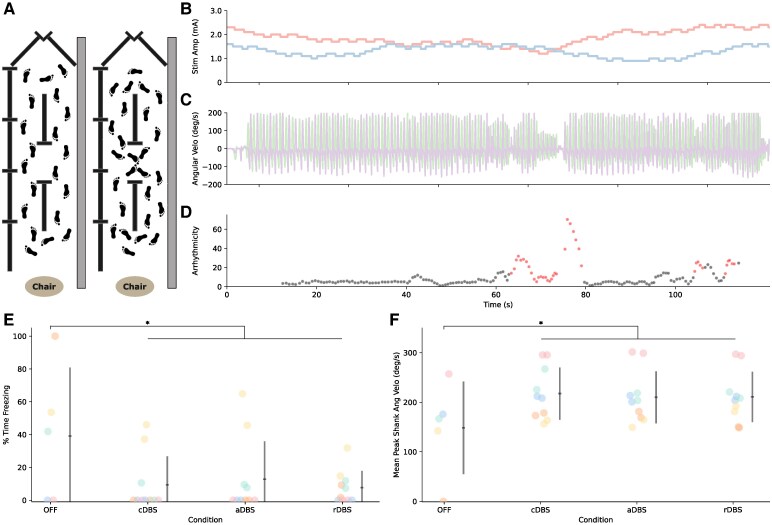
**aDBS during free walking turning and barrier course.** Drawing of a bird’s-eye view of the free walking turning and barrier course showing the ellipses and figure eights that the participant performs is shown in **A**. Example of neural aDBS during portion of the turning and barrier course for one participant (P06) is shown in **B** to **D**. Stimulation adapting independently for both STNs is shown in **B**. The left STN is shown in blue and the right STN in red. Shank angular velocity for the two legs is shown in **C**. Running arrhythmicity over the trial with freezing denoted by the red dots is shown in **D**. Percent time freezing and mean peak shank angular velocity during the turning and barrier course across stimulation conditions are shown in **E** and **F**, respectively. Individual data are shown, coloured by participant ID, along with the average and standard deviation (*N* = 5 participants and 35 total datapoints: OFF = 5, cDBS = 10, aDBS = 10, rDBS = 10; one datapoint per person for OFF DBS and two datapoints for ON DBS conditions). Linear mixed effects models found significant effects for both metrics: (Percent time freezing) *F*(3,27) = 4.73, *P* = 0.0089; (mean peak shank angular velocity) *F*(3,27) = 9.97, *P* = 0.00013. Post-hoc estimated marginal means found significant differences between OFF and DBS conditions for both metrics: (Percent time freezing) OFF-cDBS: *t* = 3. 25, *P* = 0.013, OFF-aDBS: *t* = 2.95, *P* = 0.031 and OFF-rDBS: *t* = 3.52, *P* = 0.0080, (mean peak shank angular velocity) OFF-cDBS: *t* = 5.13, *P* = 0.00010, OFF-aDBS: *t* = 4.58, *P* = 0.00050 and OFF-rDBS: *t* = 4.63, *P* = 0.00050. * indicates *P* < 0.05.

Overall, there was a significant effect of stimulation condition on percent time freezing (*F*(3,27) = 4.73, *P* = 0.0089, ${\eta_{p}}^{2}$ = 0.34; Fig. 5). Post-hoc pairwise comparisons showed significant reductions compared to OFF stimulation for aDBS (*t* = 2.95, *P* = 0.031) cDBS (*t* = 3.25, *P* = 0.013), and rDBS (*t* = 3.52, *P* = 0.0080). No difference was observed between ON stimulation conditions (aDBS–cDBS: *t* = 0.47, *P* = 0.97; aDBS–rDBS: *t* = 0.70, *P* = 0.90; cDBS–rDBS: *t* = 0.24, *P* = 1.00). Two of the three participants who demonstrated freezing in the OFF condition showed improvements in freezing for aDBS and cDBS. The two other participants showed no freezing across any conditions.

There was a significant effect of stimulation condition on mean peak shank angular velocity (*F*(3,27) = 9.97, *P* = 0.00013, ${\eta_{p}}^{2}$ = 0.53). Post-hoc pairwise comparisons showed significant improvements compared to OFF stimulation for aDBS (*t* = 4.58, *P* = 0.00050), cDBS (*t* = 5.13, *P* = 0.00010) and rDBS (*t* = 4.63, *P* = 0.00050). No difference was observed between ON stimulation conditions (aDBS–cDBS: *t* = 0.68, *P* = 0.91; aDBS–rDBS: *t* = 0.063, *P* = 1.00; cDBS–rDBS: *t* = 0.61, *P* = 0.93). All five participants showed improvements in mean peak shank angular velocity for aDBS and cDBS for all timepoints compared to OFF stimulation.

One additional participant (Participant 2) did not have values for the OFF condition for gait arrhythmicity or asymmetry due to being frozen for the entirety of the turning and barrier course. Overall, there was no significant effect of stimulation condition on gait arrhythmicity (*F*(3,26) = 0.67, *P* = 0.58, ${\eta_{p}}^{2}$ = 0.072). There was a trend toward an effect of stimulation condition on gait asymmetry (*F*(3,26.1) = 2,70, *P* = 0.067, ${\eta_{p}}^{2}$ = 0.23). Individual data are included in Supplementary Table 3.

#### Stimulation improves bradykinesia

**Figure 6:**
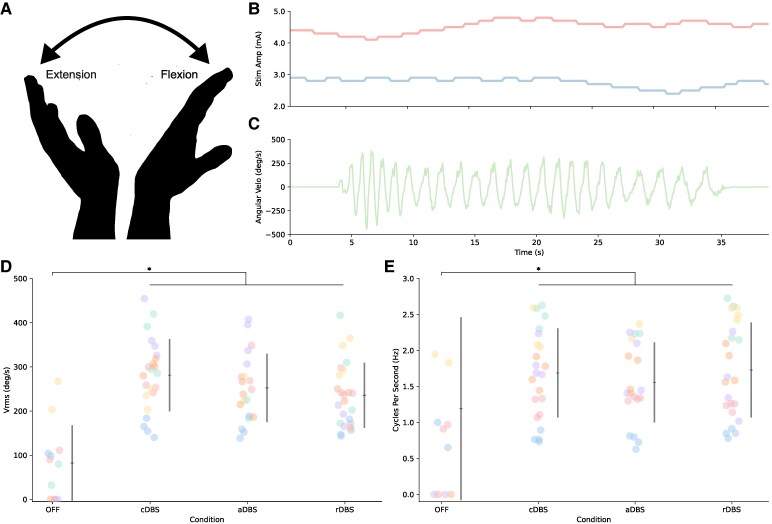
**aDBS during instrumented wrist flexion-extension task.** Depiction of the wrist flexion-extension task, a validated measure of bradykinesia is shown in **A**. Example of neural aDBS during wrist flexion-extension task for one participant (P01) is shown in **B** and **C**. Stimulation adapting independently for both STNs is shown in **B**. The left STN is shown in blue and the right STN in red. Angular velocity of the hand as it flexes and extends is shown in **C**. Root mean square velocity (*V*_rms_) and average cycles per second (Hz) during wrist flexion-extension across stimulation conditions are is shown in **D** and **E**, respectively. Individual data are shown, coloured by participant ID, along with the average and standard deviation. Data are included for right and left hand separately (*N* = 6 participants and 79 total datapoints: OFF = 12, cDBS = 23, aDBS = 20, rDBS = 24; two datapoints per person (one for each hand) for OFF DBS and four datapoints for ON DBS conditions (two for each hand). Linear mixed effects models found significant effects for both metrics: (*V*_rms_) *F*(3,70.2) = 24.4, *P* = 6.42e−11 and (number of cycles per second) *F*(3,70.2) = 4.96, *P* = 0.0035. Post-hoc estimated marginal means found significant differences between OFF and DBS conditions for both metrics: (*V*_rms_) OFF-cDBS: *t* = 8.25, *P* < 0.0001, OFF-aDBS: *t* = 7.00, *P* < 0.0001, and OFF-rDBS: *t* = 6.36, *P* < 0.0001, (number of cycles per second) OFF-cDBS: *t* = 3.37, *P* = 0.0067, OFF-aDBS: *t* = 3.26, *P* = 0.0092, and OFF-rDBS: *t* = 3.48, *P* = 0.0047. * indicates *P* < 0.05.

Overall, there was a significant effect of stimulation condition on *V*_rms_ during wrist flexion-extension (*F*(3,70.2) = 24.4, *P* = 6.42e−11, ${\eta_{p}}^{2}$ = 0.51; Fig. 6). Post-hoc pairwise comparisons showed significant improvements compared to OFF stimulation for aDBS (*t* = 7.00, *P* < 0.0001), cDBS (*t* = 8.24, *P* < 0.0001) and rDBS (*t* = 6.36, *P* < 0.0001). No difference was observed between ON stimulation conditions (aDBS–cDBS: *t* = 1.20, *P* = 0.63; aDBS–rDBS: *t* = 1.05, *P* = 0.72; cDBS–rDBS: *t* = 2.36, *P* < 0.094). The second 120-min timepoint for Participant 3 for the aDBS condition was missing for both hands due to an IMU malfunction. Participant 6 did not perform the wrist flexion-extension task with their right hand at several timepoints (120-min timepoint for cDBS and both timepoints for aDBS) due to chronic pain in their right shoulder. This pain was unrelated to the stimulation conditions. Five out of the six participants showed improvements on aDBS and cDBS compared to OFF stimulation. Individual data are included in Supplementary Table 4.

There was a significant effect of stimulation condition on the number of cycles per second (*F*(3,70.2) = 4.96, *P* = 0.0035, ${\eta_{p}}^{2}$ = 0.18). Post-hoc pairwise comparisons showed significant improvements compared to OFF stimulation for aDBS (*t* = 3.26, *P* = 0.0092), cDBS (*t* = 3.37, *P* = 0.0067) and rDBS (*t* = 3.48, *P* = 0.0047). No difference was observed between ON stimulation conditions (aDBS–cDBS: *t* = 0.014, *P* = 1.00; aDBS–rDBS: *t* = 0.11, *P* = 1.00; cDBS–rDBS: *t* = 0.10, *P* = 1.00). Individual data are included in Supplementary Table 4.

## Discussion

The current study demonstrated the feasibility, safety and tolerability of beta burst-driven aDBS in the STN for gait impairment and FOG in individuals with Parkinson’s disease. A ‘bench to bedside’ model was used in which aDBS was tested first in a harnessed stepping-in-place task, followed by a FOG-provoking free walking barrier course. All participants were able to tolerate aDBS and no adverse effects occurred that resulted in the participant being unable to tolerate the experiment. Neural aDBS significantly improved quantitative assessments of gait, such as percent time freezing, compared to OFF DBS and showed similar efficacy as traditional continuous DBS. Improvements in symptoms were also observed for quantitative assessments of bradykinesia and blinded clinical assessments of motor symptoms with the MDS-UPDRS III.

aDBS has been implemented in several different settings, including the peri-operative state,^27-29^ the laboratory with a portable or chronically implanted neurostimulator^24,30,31^ and at home.^32^ The current protocol was carried out in the laboratory setting for two primary reasons: (i) participants included in the study suffered from gait impairment and FOG and therefore posed a notable fall-risk. Previous aDBS studies demonstrating safety have either been at rest, during seated tasks, or in less impaired populations. Thus, it was crucial to first determine safety in a controlled lab environment; (ii) no currently available neurostimulator can run embedded aDBS using beta burst durations. The beta burst-driven aDBS used here required a ‘distributed-mode’ in which a computer-in-the-loop carried out the necessary signal processing for stimulation decisions.

The operation of aDBS has also differed across studies, with some employing rapid on/off fluctuations of stimulation in response to beta power using a single threshold,^27,33^ slower adaptation in response to beta power using a dual threshold,^10,24^ proportional-control^34^ or inverse algorithms based on either subcortical or cortical gamma power.^32,35,36^ Here, we implemented a single-threshold algorithm where stimulation was incremented or decremented based on the duration of the most recent ongoing beta burst. However, the goal was not necessarily to trim that most recent burst, as has been done previously in the peri-operative state^27,33^; such an approach is beyond the capabilities of the current Summit™ RC + S neurostimulator. Instead, the most recent current burst was used as an insight into the current general state of the participant, as prolonged beta bursts are related to worse gait and motor symptoms.^5,37^ Therefore, a slower ramp rate (e.g. 0.1 mA/s) was used, rather than a rapid ON/OFF change in stimulation. The slower ramp rate minimized the susceptibility to artefacts in the LFP generated by changes in stimulation.^23^

aDBS significantly reduced freezing and increased gait speed during both the harnessed stepping-in-place and free walking turning and barrier course. All five participants who showed freezing in the harnessed stepping-in-place task OFF DBS showed reductions in freezing on aDBS, and two of the three freezers in the turning and barrier course showed reductions in freezing on aDBS at both assessment timepoints, and the third showed a reduction in one of the two timepoints. Some participants still experienced some freezing regardless of stimulation condition; these were individuals with greater FOG scores (nFOG-Q ≥ 19). This lack of full amelioration could be due to several factors, such as suboptimal DBS lead location or non-dopamine contributions, such as an impaired cholinergic, noradrenergic, or other networks that would not be expected to be modified with STN DBS.^6,38^ Meanwhile, Participant 1 (a clinical non-freezer) experienced ceiling effects for each of the gait tasks, demonstrating no freezing across conditions and near normal arrhythmicity and asymmetry. aDBS would not be expected to further improve this performance. However, bradykinesia. and overall motor impairment both improved substantially on aDBS for this participant (41–81% improvement in wrist flexion-extension; 58–70% improvement in MDS-UPDRS III). It is worth noting that the majority of previous work examining the effects of aDBS, outside of the initial Little *et al*.^27^ 2013 publication, have not included any control condition where stimulation varies in a random fashion unlinked to a biomarker. Such an inclusion is crucial for better understanding the effects of aDBS. Here we found that, at the group level, the randomly adapting DBS performed similarly compared to cDBS and aDBS despite being uncoupled to the biomarker of interest. The efficacy of rDBS is likely attributed to the fact that it both used the same therapeutic window as aDBS in which it never dropped below *I*_min_, which was defined as the minimum amplitude that maintained acceptable therapeutic benefit and maintained a similar TEED. This points to the high efficacy of DBS, either static or adapting, assuming an appropriate frequency, pulse width and contact/s; any potential benefit of biologically relevant aDBS would then be additive to this already high floor.

The aDBS control policy used here did not allow stimulation to go down to 0 mA, but instead applied an *I*_min_, which was the minimum level of stimulation needed to provide acceptable therapeutic benefit. The goal of this approach was to ensure that a therapeutic level of stimulation was always maintained. Previous implementations of aDBS have shown that it may not be as effective for tremor as cDBS.^33,39^ Whereas worse bradykinesia, rigidity and gait are associated with increases in beta power, tremor shows the opposite phenomena where increased tremor is associated with attenuation of beta power.^40^ Therefore, beta-driven aDBS has previously been vulnerable to re-emergence of tremor from the positive feedback loop between tremor, beta suppression and drop in stimulation.^24,33^ However, we saw that aDBS was effective for suppression of tremor across participants, likely due to the presence of the *I*_min_ floor. aDBS improved tremor MDS-UPDRS III sub-scores 75.4 ± 23.1%, which was similar to cDBS (70.4 ± 33.6%). The use of an *I*_min_, rather than allowing stimulation to go down to 0 mA, may protect against both tremor re-emergence, as well as loss of efficacy for other motor symptoms. This is supported by our finding that randomly adapting DBS, where stimulation adapted randomly within the range of *I*_min_ to *I*_max_, also showed similar efficacy as traditional cDBS for overall motor symptoms.

A critical component of this study’s protocol was an in-depth calibration testing period to determine suitable aDBS parameters. aDBS inherently requires the setup of additional parameters compared to traditional cDBS.^22,41^ The combination of quantitative assessment of behaviour both OFF stimulation and ON DBS at different levels of stimulation amplitudes allows informed decisions of stimulation limits (i.e. *I*_min_ and *I*_max_) to ensure the maintenance of therapeutic levels of stimulation, as well as for selection of neural parameters such as the frequency band to track, value of threshold, etc. This testing allows confirmation that the signal of choice is present, modulates with stimulation, particularly in the range between *I*_min_ and *I*_max_, and is not corrupted by any sources of artefact. We carried out this testing both at rest and during movement to determine neural thresholds that were robust across movement states.^42^

aDBS did not exhibit superior performance compared to cDBS. This is not surprising given the acute protocol (20–120 min of stimulation) and effectiveness of cDBS, and follows previous in-lab results.^33,36,43^ aDBS may require longer durations to establish superiority to cDBS, as the mechanisms of biologically relevant neuromodulation likely operate on longer timescales. This is supported by a recent publication demonstrating superiority for aDBS when delivered over days at home^32^; however, in that study aDBS used a 15% increase in average TEED compared to cDBS which may also explain its superiority. In the current study, TEED was purposefully matched across conditions (see Supplementary Materials) to isolate the effect of adapting stimulation in a biologically informed fashion without any confound of TEED differences.

### Limitations

Only seven individuals were tested due to the discontinuation of the Summit™ RC + S. However, this represents the largest cohort to date with the Summit™ RC + S, and no neurostimulator currently available can use beta burst duration as an input for aDBS. aDBS requires a sense-friendly configuration in which the recording contacts must flank the stimulation contact/s.^22^ In our cohort, four STNs across three participants required altering the clinical stimulation contacts used to ensure a sense-friendly configuration. All three of these participants were IPG re-implants, and thus further along in their disease progression and required higher stimulation amplitudes and double monopolar configurations. Typically, patients newer to DBS are more likely to only require single monopolar configurations, and thus more likely to have sense-friendly configurations. Three STNs across three participants were unusable for sensing due to either stimulation-related artefact or insufficient modulation of beta within the therapeutic window; therefore, there is a question of the widespread feasibility of independent bilaterally driven aDBS. There are also additional considerations for the most suitable candidates for aDBS for FOG. FOG is a heterogeneous condition that is unlikely to have a single treatment for all patients given the wide variety of contributing factors that can result in FOG. For instance, FOG unresponsive to levodopa is unlikely to be improved by any form of STN DBS, regardless of continuous or adaptive. On the other end of the spectrum, it may be challenging to evaluate adaptive DBS for gait impairment or FOG in the clinical context for individuals with mild levels of gait impairment/FOG where it can be difficult to reliably elicit FOG to ascertain efficacy of therapy. However, even these individuals would still be potentially suitable for beta burst-driven adaptive DBS as it would still be expected to improve the other cardinal motor signs of PD which can be more easily evaluated, as was seen with P01. It is worth noting that the current study only tested the acute effects of aDBS. It is unknown from the current study whether there would be additional benefit if on the controller for longer than the 2 h tested here. aDBS also requires more extensive setup compared to cDBS.^41^ Larger future trials should give more insight into whether this trade-off is worth potential additional benefit from aDBS. Lastly, the beta burst-driven aDBS control policy here required a computer-in-the-loop distributed system and thus is limited to a research laboratory setup. Translation of such an approach would require a system capable of embedded aDBS. However, beta burst duration is correlated with beta power, and thus available systems such as the Percept PC/RC, AlphaDBS, etc., which can run embedded aDBS based on power, could offer similar effects.

## Conclusions

This is the first study to demonstrate the feasibility, safety and tolerability of beta burst-driven adaptive DBS in the STN for freezing of gait in Parkinson’s disease in the largest cohort to date using the investigative Summit™ RC + S neurostimulator. Neural aDBS improved quantitative metrics of gait compared to OFF DBS and provided similar efficacy as traditional cDBS. These improvements were maintained for all cardinal motor signs, including tremor. The current findings pave the way for future long-term testing of aDBS outside of the laboratory, even in participants with FOG and more severe motor impairments.

## Supplementary Material

fcaf266_Supplementary_Data

## Data Availability

Data will be made available by the corresponding author upon reasonable request. Analysis code is available at the following github repository: https://github.com/bronte-stewart-lab/AdaptiveDBSAnalysis.
