# Supplementary material for: Beta burst-driven adaptive deep brain stimulation for gait impairment and freezing of gait in Parkinson’s disease
Source: Brain Commun. 2025 Jul 9;7(4):fcaf266. doi: 10.1093/braincomms/fcaf266 (PMC12268161; doi:10.1093/braincomms/fcaf266)
Supplement: fcaf266_Supplementary_Data [file fcaf266_supplementary_data.zip › Supplementary_Materials.pdf]

Supplementary Materials

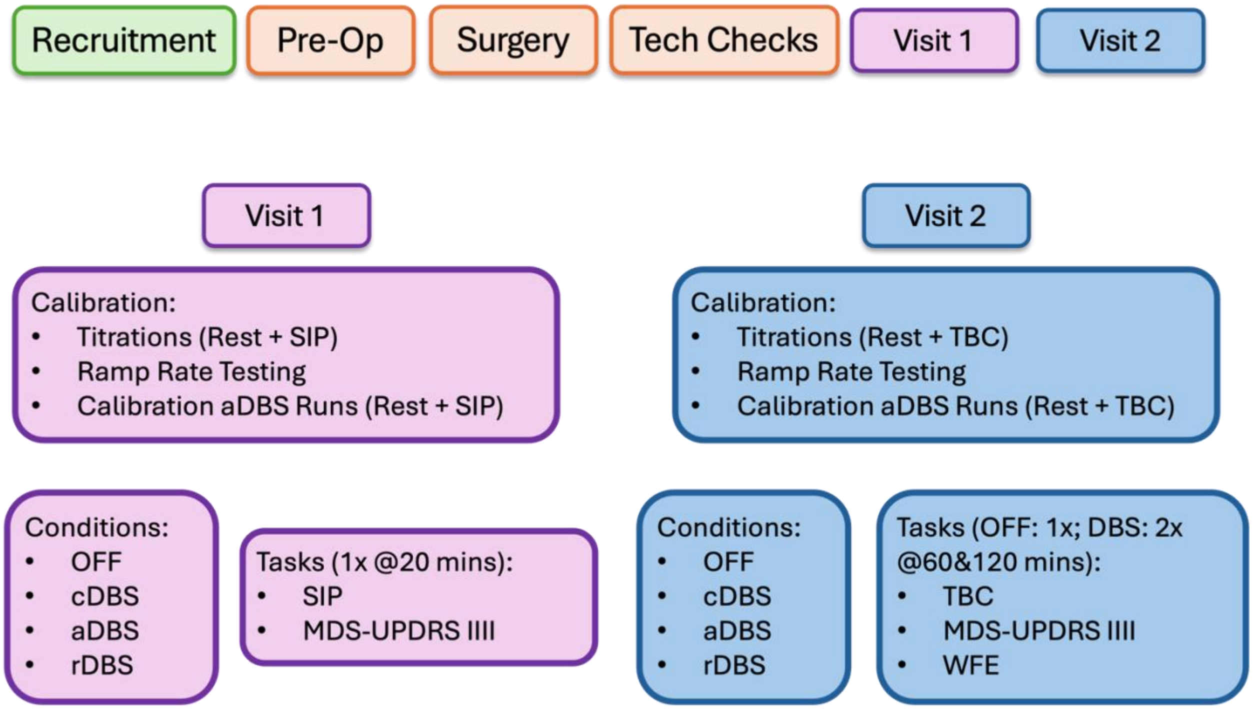

Supplementary Figure 1. Schematic of clinical trial timeline and protocol for the two visits. SIP: Stepping-In-Place Task; MDS-UPDRS III: Movement Disorders Society-Unified Parkinson's Disease Rating Scale Part III; TBC: Turning and Barrier Course; WFE: Wrist Flexion-Extension Task; cDBS: continuous Deep Brain Stimulation; aDBS: adaptive Deep Brain Stimulation; rDBS: Random adaptive Deep Brain Stimulation

Parameters for Other DBS Conditions

The calibration runs were also used to establish the parameters used for the cDBS and rDBS conditions. The goal of the cDBS was to match the total electrical energy delivered (TEED) during aDBS. Therefore, the average observed stimulation intensity during the calibration aDBS run was used as the stimulation intensity for the cDBS condition. The goal of the rDBS was to match both the TEED and the general overall pattern of stimulation variation, without linking the

stimulation adaption to beta burst durations. Thus, the observed modulation of stimulation amplitude during the calibration aDBS run was randomly shuffled for the rDBS condition.

Local Field Potential Data Acquisition

LFPs were defined from the difference between the two sensing contacts, which serves to further reduce stimulation-related artifact through common mode rejection[1,2]. Active recharge was enabled, which reduced stimulation- and ECG-related artifacts. Data were streamed off the device via the CTM in packets averaging 50 ms. Mode 4 was chosen for data streaming, which allowed faster transmission of packets compared to the alternative available Mode 3 on the Summit device. Mode 4 required the CTM to be in closer proximity to the IPG compared to Mode 3. A cloth sleeve held the CTM and was pressed against the IPG using the strap from the chest IMU.

A custom graphical user interface (GUI) application was used for configuring and streaming data based on a C# API provided by Medtronic as part of a research development kit[3].

Kinetic and Kinematic Data Analysis

Stepping-in-Place:

The stride time begins with initial contact of one foot with the ground and ends just prior to contact of the same foot with the ground. Swing time was identified as the duration between when one foot left the force plate and when the same foot contacted the force plate again. Swing times and stride times were used to calculate asymmetry and arrhythmicity

Turning and Barrier Course:

The beginning of the swing phase was denoted by the positive slope zero crossing; the end of the swing phase was denoted by the subsequent negative zero crossings; and peak shank angular velocities were identified as the first positive peak following the beginning of the swing phases. The time between subsequent zero crossings of the same leg denoted forward swing phase and the time between consecutive peak shank angular velocities was used to calculate stride times. The peaks of shank angular velocity were used to identify strides to avoid difficulty of discerning heel strikes in PD[4]. Peaks were marked as steps only if they exceeded a minimum threshold 10 deg/s for TBC. Swing angular range was calculated by integrating the sagittal angular velocity curve during swing phase.

Synchronization of Local Field Potential and Kinematic Data

Synchronization of neural, kinetic, kinematic, and video recordings was done using internal and external instruments using a data acquisition interface (Power1401) and Spike software (version 2.7, Cambridge Electronic Design, Ltd., Cambridge, England). The APDM IMU system sent a TTL pulse at the start of recording. Bertec force plate data were synchronized using two transient pulses to the force plate simultaneously captured by an accelerometer placed on the force plate connected to the data acquisition interface. Neural data were synchronized using either a 20 Hz 1.5 mA stimulation train for a few seconds in the OFF stimulation condition, or by transiently switching from 140 Hz to 20 Hz and back to 140 Hz in the ON stimulation conditions. Surface electrodes were attached to the skin over the wires of the IPG which connected to the data acquisition interface and allowed detection of the stimulation-induced artifact when switching to 20 Hz.

Total Electrical Energy Delivered

The Total Electrical Energy Delivered (TEED) was calculated using the following equation[5]:

$$P_W = I_{(A)}^2 \times pw_{sec} \times f_{(Hz)} \times R_\Omega$$

P = power, W = watts, I = current, A = amps, pw = pulse width, f = frequency, R = resistance, and Ω = ohms. In the case of a double monopolar configuration, the TEED was calculated separately for each active contact and then summed. TEED was calculated in one second intervals (TEED (1 s)) and averaged across the trial.

Lead Reconstruction

Preoperative T_1 and T_2 MRI scans and postoperative CT scans were acquired as part of the standard Stanford clinical protocol[6]. Location of DBS leads was determined by the Lead-DBS toolbox[7]. Postoperative CT scans and preoperative T_2 scans were co-registered to preoperative T_1 scans, which were then normalized into MNI space using SPM12 (Statistical Parametric Mapping 12; Wellcome Trust Centre for Neuroimaging, UCL, London, UK) and Advanced Normalization Tools[8]. DBS electrode localizations were then corrected for brain-shift in the postoperative CT scan[9]. DBS electrodes were then localized in template space using the PaCER algorithm[10] and projected onto the DISTAL Atlas to visualize overlap with the STN[11].

Supplementary Results

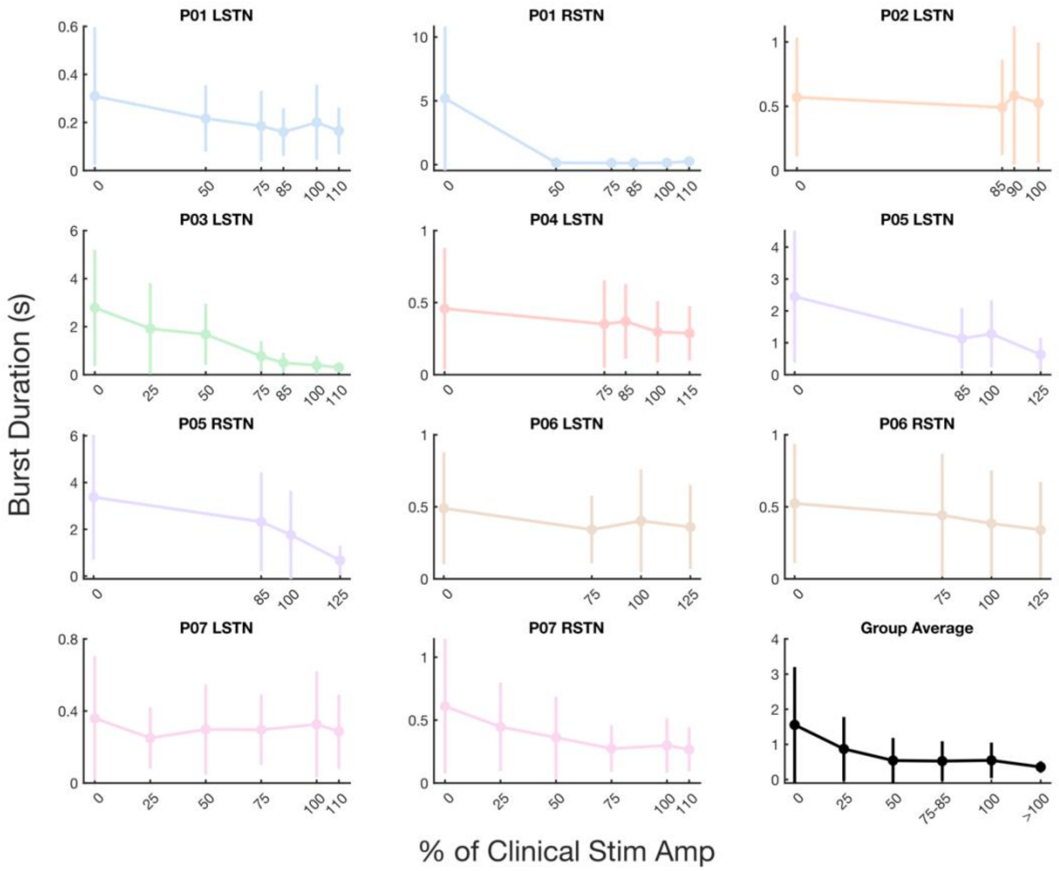

Supplementary Figure 2. Comparison of burst duration across randomized stimulation amplitudes during the calibration for the stepping-in-place task. Each subplot depicts an STN from a given participant. The group average is shown in the bottom right. The marker depicts the group average and the bars represent the standard deviation. (N=7 participants, 11 STNs and 56 total datapoints. A Linear mixed effects model found significant effect of stimulation on average beta burst duration: t=4.176, p=1.4e-4.

Comparison of Total Electrical Energy Delivered (TEED)

There was no significant effect of stimulation condition on TEED during the SIP task ($F(2,33)=0.0065$, $p=0.99$, $\eta_p^2=0$), TBC task ($F(2,52.5)=0.067$, $p=0.227$, $\eta_p^2=0.003$), or rWFE task ($F(2,62)=0.036$, $p=0.96$, $\eta_p^2=0.001$).

Supplementary Table 1. Effect of Stimulation on MDS-UPDRS III Sub-scores

MDS-UPDRS III Sub-score	OFF vs. aDBS	OFF vs. cDBS	OFF vs. rDBS
Bradykinesia	$t = 9.70, p < 0.0001$	$t = 9.59, p < 0.0001$	$t = 9.08, p < 0.0001$
Tremor	$t = 9.39, p < 0.0001$	$t = 8.64, p < 0.0001$	$t = 8.37, p < 0.0001$
Rigidity	$t = 6.25, p < 0.0001$	$t = 6.60, p < 0.0001$	$t = 7.21, p < 0.0001$

Supplementary Table 2. MDS-UPDRS III ON-stimulation Sub-score Comparisons

MDS-UPDRS III Sub-score	aDBS vs. cDBS	aDBS vs. rDBS	cDBS vs. rDBS
Total	$t = 0.023, p = 1.00$	$t = 0.19, p = 1.00$	$t = 0.21, p = 1.00$
Bradykinesia	$t = 0.12, p = 1.00$	$t = 0.74, p = 0.90$	$t = 0.58, p = 0.94$
Tremor	$t = 0.85, p = 0.83$	$t = 1.16, p = 0.65$	$t = 0.31, p = 0.99$
Rigidity	$t = 0.40, p = 0.98$	$t = 1.09, p = 0.70$	$t = 0.69, p = 0.90$

Supplementary Table 3. Individual Gait Metrics Across Visits

ID	Condition	SIP		TBC (60 mins)		TBC (120 mins)	
		Arrhythmicity	Asymmetry	Arrhythmicity	Asymmetry	Arrhythmicity	Asymmetry
01	OFF	9.1	17.7	7.5	7.5	-	-
	aDBS	6.1	3.1	7.7	6.0	7.6	7.6
	cDBS	5.9	6.9	6.5	6.5	7.4	2.7
	rDBS	7.6	7.6	6.3	7.3	7.2	6.3
02	OFF	-	-	-	-	-	-
	aDBS	13.6	23.8	7.3	4.2	7.7	3.0
	cDBS	8.3	22.8	7.8	7.9	9.1	9.2
	rDBS	11.8	24.5	19.3	22.1	22.4	35.9
03	OFF	-	-	24.6	18.3	-	-
	aDBS	149.2	17.1	11.8	20.1	11.2	19.5
	cDBS	190.4	11.4	11.6	12.5	18.1	7.4
	rDBS	166.4	8.6	15.1	22.5	15.1	11.9
04	OFF	204.4	16.7	5.6	7.2	-	-
	aDBS	4.7	3.8	6.9	7.2	6.5	10.5
	cDBS	4.5	3.0	6.1	10.2	6.7	3.1
	rDBS	4.4	3.0	5.8	9.2	5.6	8.3
05	OFF	-	-	-	-	-	-
	aDBS	180.8	7.6	-	-	-	-
	cDBS	131.5	21.6	-	-	-	-
	rDBS	-	-	-	-	-	-
06	OFF	19.1	8.9	34.1	1.9		
	aDBS	16.3	0.4	41.5	3.7	28.5	5.5
	cDBS	6.5	3.8	37.6	5.0	28.2	2.1
	rDBS	5.6	3.8	26.0	4.3	22.8	9.0
07	OFF	10.5	13.7	-	-	-	-
	aDBS	3.9	4.4	-	-	-	-
	cDBS	3.6	6.5	-	-	-	-
	rDBS	3.5	2.1	-	-	-	-

*Participants 05 and 07 did not complete the TBC task. Dashed lines indicate the participant was frozen for the entirety of the trial or did not complete the trial. SIP: Stepping-In-Place Task. TBC: Turning and Barrier Course.

Supplementary Table 4. Individual WFE Metrics

ID	Condition	Visit 2 (60 mins)				Visit 2 (120 mins)			
		V_{RMS}		# Cycles/Second		V_{RMS}		# Cycles/Second	
		L	R	L	R	L	R	L	R
01	OFF	98.6	104.1	0.65	1.00	-	-	-	-
	aDBS	159.2	188.5	0.80	0.81	138.8	152.4	0.63	0.73
	cDBS	154.7	165.0	0.76	0.76	140.1	183.9	0.73	0.89
	rDBS	156.7	181.5	0.73	0.91	1447.1	143.3	0.85	0.85
02	OFF	0	0	0	0	-	-	-	-
	aDBS	238.0	214.7	1.41	1.92	266.6	186.3	1.46	1.86
	cDBS	280.0	317.9	1.45	1.78	300.6	306.4	1.60	1.91
	rDBS	250.1	241.8	1.56	1.93	222.7	162.2	1.46	2.10
03	OFF	32.2	80.3	3.42	3.57	-	-	-	-
	aDBS	183.6	225.0	2.24	2.23	-	-	-	-
	cDBS	294.0	391.1	2.30	2.47	285.6	419.6	2.58	2.63
	rDBS	172.5	309.9	2.17	2.15	202.3	416.7	2.61	2.72
04	OFF	111.1	90.0	0.91	0.97	-	-	-	-
	aDBS	277.2	348/7	1.32	1.34	249.1	268.4	1.30	1.35
	cDBS	241.7	258.5	1.11	1.07	253.2	299.7	1.33	1.32
	rDBS	237.3	297.9	1.14	1.26	241.6	240.4	1.26	1.23
05	OFF	0	0	0	0	-	-	-	-
	aDBS	395.4	407.4	1.41	2.10	337.2	306.4	1.45	2.25
	cDBS	325.9	359.2	1.69	1.79	347.0	454.4	1.67	2.24
	rDBS	171.7	213.4	1.34	2.26	193.1	165.2	1.02	1.63
06	OFF	267.3	203.5	1.95	1.83	-	-	-	-
	aDBS	278.5	-	2.36	-	223.3	-	2.16	-
	cDBS	235.6	202.5	2.05	2.08	249.7	-	2.59	-
	rDBS	348.6	365.2	2.60	2.59	280.8	292.7	2.49	2.42

Supplementary References

1. Stanslaski, S. *et al.* A Chronically Implantable Neural Coprocessor for Investigating the Treatment of Neurological Disorders. *IEEE Trans. Biomed. Circuits Syst.* **12**, 1230–1245 (2018).

2. Stanslaski, S. *et al.* Design and Validation of a Fully Implantable, Chronic, Closed-Loop Neuromodulation Device With Concurrent Sensing and Stimulation. *IEEE Trans. Neural Syst. Rehabil. Eng.* **20**, 410–421 (2012).

3. Petrucci, M. N. *et al.* A Closed-loop Deep Brain Stimulation Approach for Mitigating Burst Durations in People with Parkinson's Disease. *Proceedings of the Annual International Conference of the IEEE Engineering in Medicine and Biology Society, EMBS* **2020-July**, 3617–3620 (2020).
4. O'Day, J. *et al.* The turning and barrier course reveals gait parameters for detecting freezing of gait and measuring the efficacy of deep brain stimulation. *PLoS ONE* **15**, (2020).
5. McAuley, M. D. Incorrect calculation of total electrical energy delivered by a deep brain stimulator. *Brain Stimulation* **13**, 1414–1415 (2020).
6. Brontë-Stewart, H., Louie, S., Batya, S. & Henderson, J. M. Clinical motor outcome of bilateral subthalamic nucleus deep-brain stimulation for Parkinson's disease using image-guided frameless stereotaxy. *Neurosurgery* **67**, 1088–1093; discussion 1093 (2010).
7. Horn, A. *et al.* Lead-DBS v2: Towards a comprehensive pipeline for deep brain stimulation imaging. *NeuroImage* **184**, 293–316 (2019).
8. Avants, B. B. *et al.* A Reproducible Evaluation of ANTs Similarity Metric Performance in Brain Image Registration. *Neuroimage* **54**, 2033–2044 (2011).
9. Horn, A. & Kühn, A. A. Lead-DBS: a toolbox for deep brain stimulation electrode localizations and visualizations. *Neuroimage* **107**, 127–135 (2015).
10. Husch, A., V. Petersen, M., Gemmar, P., Goncalves, J. & Hertel, F. PaCER - A fully automated method for electrode trajectory and contact reconstruction in deep brain stimulation. *NeuroImage: Clinical* **17**, 80–89 (2018).
11. Ewert, S. *et al.* Toward defining deep brain stimulation targets in MNI space: A subcortical atlas based on multimodal MRI, histology and structural connectivity. *NeuroImage* **170**, 271–282 (2018).
