# Supplementary material for: Beta burst-driven adaptive deep brain stimulation for gait impairment and freezing of gait in Parkinson’s disease
Source: Brain Commun. 2025 Jul 9;7(4):fcaf266. doi: 10.1093/braincomms/fcaf266 (PMC12268161; doi:10.1093/braincomms/fcaf266)
Supplement: fcaf266_Supplementary_Data [file fcaf266_supplementary_data.zip › Supplementary Video 1 Legend.pdf]

Supplementary Video 1. Example of ramp-rate artifact in LFP. (Top) Randomized changes in stimulation amplitude during ramp rate testing. (Bottom) Corresponding LFP data. Increments/decrements of stimulation cause a transient artifact in the LFP.
